# Prediction of Post-operative Visual Deterioration Using Visual-Evoked Potential Latency in Extended Endoscopic Endonasal Resection of Craniopharyngiomas

**DOI:** 10.3389/fneur.2021.753902

**Published:** 2021-12-03

**Authors:** Xiaorong Tao, Xiaocui Yang, Xing Fan, Hao You, Yanwen Jin, Jiajia Liu, Dongze Guo, Chuzhong Li, Hui Qiao

**Affiliations:** ^1^Department of Neurophysiology, Beijing Neurosurgical Institute, Capital Medical University, Beijing, China; ^2^Department of Neurosurgery, Beijing Tiantan Hospital, Capital Medical University, Beijing, China

**Keywords:** craniopharyngioma, extended endoscopic endonasal approach, optic nerves, visual evoked potential (VEP), post-operative visual deterioration

## Abstract

**Background:** The current study aimed to investigate the predictive value of visual-evoked potential (VEP) latency for post-operative visual deterioration in patients undergoing craniopharyngioma resection via extended endoscopic endonasal approach (EEEA).

**Methods:** Data from 90 patients who underwent craniopharyngioma resection *via* EEEA with intraoperative VEP monitoring were retrospectively reviewed. P100 latency was compared between patients with and without post-operative visual deterioration, and the threshold value of P100 latency for predicting post-operative visual deterioration was calculated by the receiver operating characteristic curve analysis. In addition, other potential prognostic factors regarding post-operative visual outcomes were also analyzed by multivariate analysis.

**Results:** Patients with post-operative visual deterioration showed a significantly longer VEP latency than those without (*p* < 0.001). An extension over 8.61% in VEP latency was identified as a predictor of post-operative visual deterioration (*p* < 0.001). By contrast, longer preoperative visual impairment duration and larger tumor volume were not significant predictors for post-operative visual deterioration.

**Conclusions:** The current study revealed that intraoperative VEP monitoring in EEEA is effective for predicting post-operative visual deterioration, and an extension over 8.61% in VEP latency can be used as a critical cut-off value to predict post-operative visual deterioration.

## Background

Craniopharyngioma represents around 1.2–4.6% of all intracranial tumors. It is originated from the remnant of Rathke's pouch and histologically benign (Grade I according to the 2016 World Health Organization Classification). Nowadays, radical resection is still the preferred treatment for craniopharyngioma. However, due to its complex anatomical relationships with optic nerves and chiasm, pituitary stalk, gland and hypothalamus, complete resection is usually difficult, and the risk of relevant post-operative complications is quite high (1, 2). Microscopic transcranial surgical approaches were considered as classic operative strategy for craniopharyngiomas, but in recent decades, extended endoscopic endonasal approach (EEEA) has revolutionized the surgical treatment of craniopharyngiomas. It has proved that the EEEA could provide the shortest direct corridor and also maximal exposure without any brain retraction. Although the EEEA could provide an improved visual field of the optic nerves and chiasm, and hypothalamus, injury of optic pathways is still the most common surgical complication (3–5).

Over the past decades, intraoperative neurophysiological monitoring (IONM) has become a crucial component for modern neurosurgery. Specific to visual function protection, visual-evoked potential (VEP) is proved to be an effective modality for reflecting the integrity of visual pathway from retina to pulvinar cortex, and has been applied to surgeries with the risk of visual pathway damage (6–9). To our knowledge, in the research field of VEP monitoring, most studies have focused on the predictive value of VEP amplitude for post-operative visual deterioration. By contrast, few studies have been designed to identify the relationship of VEP latency with post-operative visual outcome (10). In the current study, we aimed to investigate the predictive value of P100 latency of VEP for post-operative visual deterioration in patients undergoing EEEA, and to attempt to calculate an appropriate threshold of P100 latency prolongation to avoid post-operative visual deterioration.

## Methods

### Patients

Data of 124 craniopharyngioma patients undergoing EEEA from June, 2019 to February, 2021 at Beijing Tiantan Hospital were retrospectively reviewed. The inclusion criteria were as follows: (i) aged from 16 to 65 years old; (ii) received VEP monitoring. The exclusion criteria were as follows: (i) severe visual impairment preoperatively (blindness or severe visual field defect); (ii) with a pathological diagnosis not consistent with craniopharyngioma. This study was approved by the ethics committee of Beijing Tiantan Hospital. All patients provided informed consent for participation in the study.

### Anesthesia

Total intravenous anesthesia was induced by propofol (2–2.5 mg/kg) and sufentanil (0.3–0.4 μg/kg) and was maintained by continuous infusion of propofol (4–12 mg/kg/h) and remifentanil (0.05–0.2 μg/kg/min). The BIS values were maintained between 40 and 60 using a dual spectral index sensor (VISTA monitoring system, Massachusetts, USA). The patient's heart rate, blood pressure, blood oxygen, and carbon dioxide levels were continuously monitored during surgery.

### Neurophysiological Monitoring

**Figure 1:**
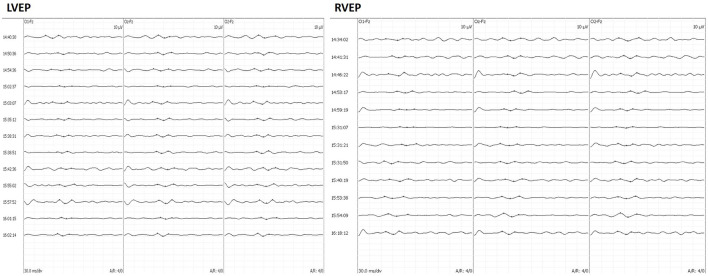
A 49-year-old man complained of vision loss for 6 months. Intraoperatively, the patient underwent craniopharyngioma resection through EEEA with VEP monitoring. The left VEP latency was prolonged until the end of surgery, and the right VEP latency was prolonged intraoperatively and returned to baseline levels by the end of surgery. Follow-up at 3 months after surgery showed a decrease in visual acuity in the left eye and no change in the right eye, respectively. EEEA, extended endoscopic endonasal approach; VEP, visual-evoked potential.

A monitoring protocol including ERG and VEP monitoring was applied to all patients enrolled in the study. A photostimulation device consisting of a white LED (NimEclipse; Medtronic) served as the stimulator, flashing at a frequency of 0.7–1.2 Hz. The brightness of the LED was set to 500–10,000 Lx, and the duration of each stimulus was 84 ms. Goggles were applied to both closed eyelids. The needle electrodes for ERG were placed 2 cm from the external canthus. For VEP monitoring, the recorded sites were O1, O2, and Oz, and the reference electrodes were placed on Fz, according to the International EEG Electrode Placement 10–20 System Standard, with bilateral recording using monocular stimulation. The recording electrodes were corkscrew electrodes. The bandpass filter was set from 1 to 100 Hz and stacked <50 times. The analysis times were 300 ms. VEP data were collected and evaluated by one of the two experienced IONM technicians with corresponding equipment (NIM-ECLIPSE Nerve Monitoring System, Medtronic, Medtronic, Minneapolis, MN, USA). VEP were recorded once after anesthesia and recorded at least three times to ensure repeatable waveforms. The stable VEP waveform after opening the dura was considered as the baseline. For uncoupling of the optic canal and removal of the tumor, intraoperative VEP monitoring was performed every 2 min until the end of the skull base reconstruction. Intraoperatively, we focused on the latency of the large positive peak (P100) at around 100 ms. A warning is purely given according to the presence of P100 latency prolongation. The neurosurgeon will change the surgical strategy, such as stopping the retraction of the optic nerve or adjusting the filler position, to restore the P100 latency. The degree of P100 latency prolongation was calculated by the following formula: (post-operative latency—baseline)/baseline^*^100%. Figure 1 shows VEP waveforms acquired during the surgery.

### Clinical Assessment and Follow-Up

Preoperative computed tomography and magnetic resonance imaging (MRI) were performed for preoperative evaluation and used to examine the tumor volume. Tumor volume was calculated using the following formula: volume = 4/3π (a/2 × b/2 × c/2), where a, b, and c represent the diameter dimensions of the three. Post-operative MRI was performed within 2 weeks after surgery to confirm the extent of tumor removal. Gross total removal was defined as resection without visible remnant tumor according to intraoperative assessment and post-operative MRI.

All patients underwent best-corrected visual acuity (BCVA) examinations preoperatively and at least 3 months after surgery. BCVA (normal ≥ 1.0) was assessed by a trained and certified research optometrist using a logarithmic visual acuity (VF) scale at a distance of 5 m under standard illumination. BCVA in decimal units was converted to the logarithm of the minimum angle of resolution (logMAR) for analysis. A change of <1 line was defined as no deterioration in visual acuity, and a loss of at least one line was defined as deterioration in visual acuity.

Adhesion forces between neoplasms and optical nerves were classified into two categories according to the intraoperative findings of the surgeon: (1) no or loose adhesion if the tumor can be easily separated from the optic apparatus with gentle blunt dissection using dissectors; or (2) tight adhesion if separation of the tumor needed sharp dissection using scissors (11, 12).

### Statistical Analysis

All statistical analyses were performed with the SPSS software version 22.0 (SPSS Inc., Chicago, Illinois, USA) and GraphPad Prism (version 8.0.1 for Windows, GraphPad Software, San Diego, California, USA). Normally distributed samples were expressed as the mean ± standard deviation, median [with interquartile range (IQR)] for non-normally distributed samples. The degree of P100 latency prolongation between patients with post-operative visual deterioration and those without was compared by the Mann–Whitney *U*-test. A *p* < 0.05 was considered statistically significant.

Receiver operating characteristic curve analysis was used to calculate the optimal threshold for the degree of prolongation of P100 latency to avoid post-operative visual dysfunction. A chi-square test was used to compare patients with a prolonged P100 latency less than or greater than the threshold. Univariate logistic regression was used to describe the correlation between the parameters of interest and post-operative visual function deterioration.

## Results

### Clinical and Demographic Characteristics of Craniopharyngiomas Patients

Ultimately, 90 patients (48 men and 42 women) were enrolled. The mean age of the enrolled patients was 42 years (range, 16–65). Craniopharyngiomas of adamantinomatous type were histopathologically diagnosed in 48 cases (73.8%), whereas papillary type was identified in 17 cases (26.2%). Among the 90 tumors, 66 (73.3%) were primary craniopharyngiomas and 24 (26.7%) were recurrent craniopharyngiomas. The median duration of preoperative visual impairment was 4 months (range, 1–72 months). The median tumor volume was 7.59 cm^3^ (3.32–17.80 cm^3^). Craniopharyngiom were loose or unattached in 52 cases (57.8%) and adherent tight in 38 cases (42.2%). Of the 10 cases with residual tumors, six were observed without further treatment, and four were treated with gamma knife after 3 months.

### P100 Latency of VEP

Reproducible and stable VEP monitoring results were obtained in all 180 (100%) eyes of the enrolled 90 patients. The preoperative P100 latency was 101.50 ms (94.00–109.50 ms) and 101 ms (90.50–107.50 ms) post-operatively. The post-operative P100 latency was significantly shorter than the preoperative one, with a statistically significant difference (*p* < 0.001, Wilcoxon test).

### Prolonged P100 Latency and Post-operative Visual Deterioration

**Figure 2:**
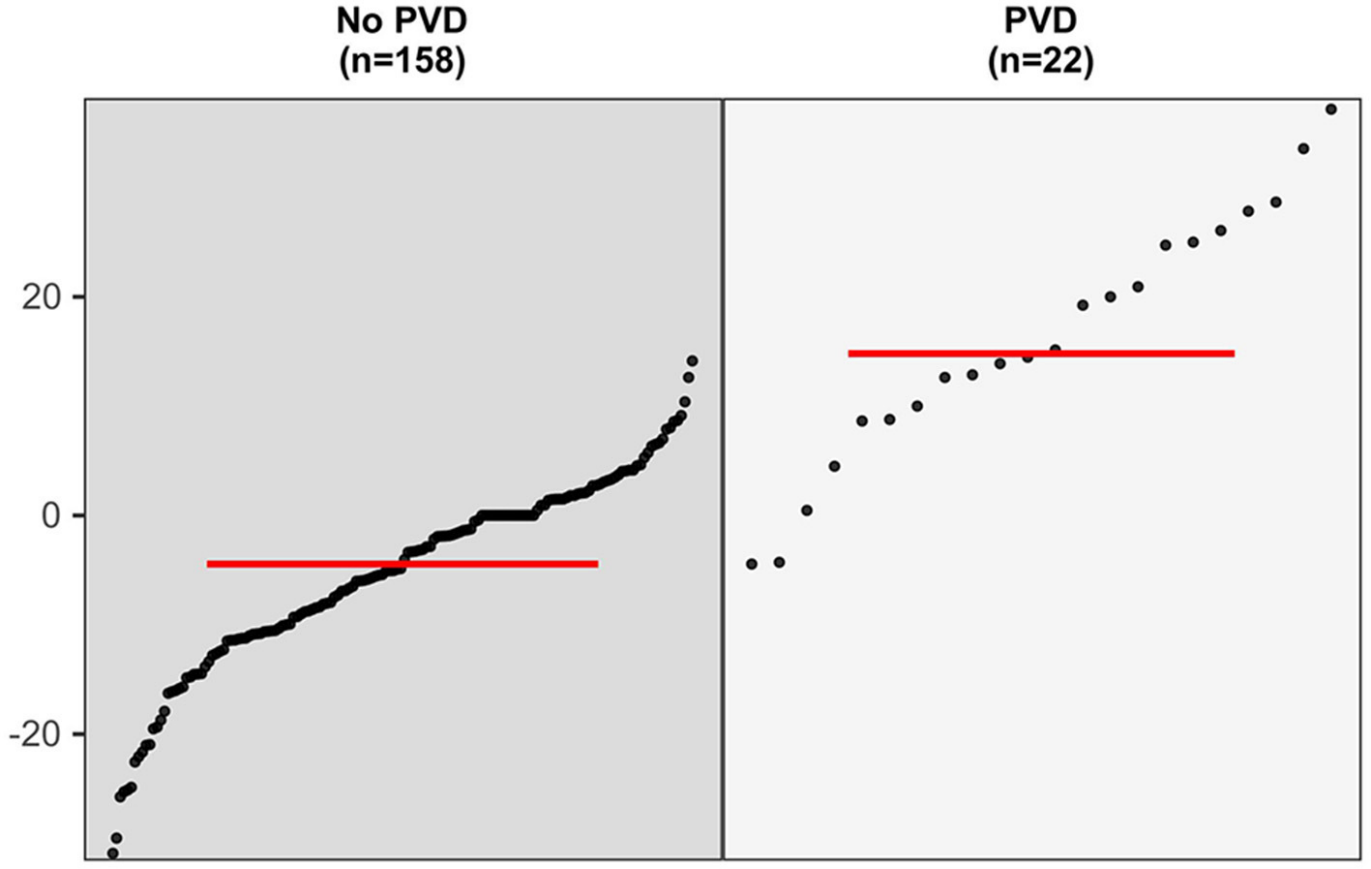
The comparison in the degree of P100 latency prolongation between eyes with post-operative visual deterioration and those without (Mann–Whitney *U*-test, *p* < 0.001).

In the 180 eyes of 90 patients included in this study, the degree of P100 latency prolongation was 14.81% (8.75–25.27%) in the 22 (12.22%) eyes with post-operative visual deterioration and −4.45% (−10.84 to −1.41%) in 158 (87.78%) eyes without post-operative visual deterioration. The degree of P100 latency prolongation was significantly higher in patients with post-operative visual deterioration than in those without deterioration (Mann–Whitney *U*-test, *p* < 0.001, Figure 2).

**Figure 3:**
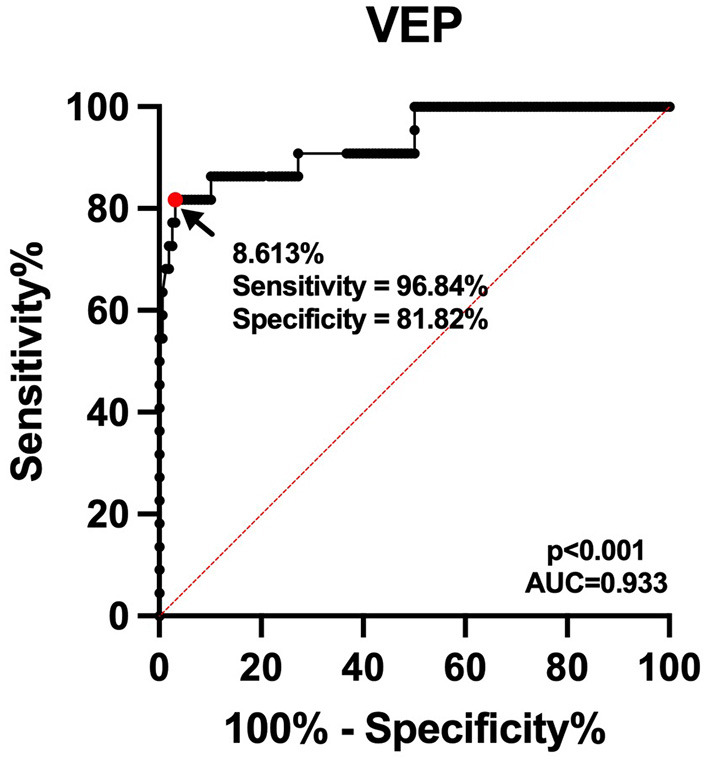
Receiver operating characteristic curve for the prediction of post-operative visual deterioration. The continuous line shows the sensitivity, and dashed line shows identity. The area under the curve is 0.933.

The optimal threshold value for avoidance of post-operative visual deterioration was 8.61% (AUC = 0.933, 95% CI 0.869–0.997*, p* < 0.001, Figure 3). The 23 eyes had post-operative P100 latency prolongation ≥8.61%, 15 eyes had no changes, and 142 eyes had P100 latency prolongation <8.61% or varying degrees of shortening. In 23 eyes with prolonged P100 latency, 18 eyes showed post-operative visual deterioration, and five eyes did not show those. Out of 157 eyes with shortened or unchanged P100 latency, four eyes showed post-operative visual deterioration, and 153 eyes did not show post-operative visual deterioration. Patients with a prolonged post-operative P100 latency ≥8.61% had a significantly higher risk of worsening post-operative visual function (chi-square test, *p* < 0.001, Table 1).

**Table 1:** Relationship between intraoperative P100 latency prolongation and post-operative visual outcome.

**Visual deterioration (*n*)**	**P100 latency prolongation**		**Total** **(*N* = 180)**	***P*-Value**
	**Yes**	**No**		
Yes	18	4	22	<0.001
No	5	153	158	

### Other Potential Predictors

Logistic regression analysis was performed to further investigate other potential predictors for post-operative visual deterioration. Variables such as gender, age, whether recurrence, preoperative visual impairment duration, pathology type, tumor volume, the degree of adhesion, and the extent of resection were analyzed. As in all 70 patients with prolonged post-operative P100 latency <8.61% for both eyes, post-operative visual deterioration was not observed, and prolonged post-operative P100 latency was not applied to logistic regression analysis; so was the extent of resection (in all 10 patients who received total resection, post-operative visual deterioration was not observed). The results of univariate logistic regression analysis showed that none of the above variables could be used to predict post-operative visual deterioration (Table 2).

**Table 2:** Univariate logistic regression analysis of potential predictors.

**Variable**	**Post-operative visual deterioration**		
	**OR**	**95% CI**	***P*-Value**
Gender	0.51	0.16–1.65	0.26
Age	1.02	0.97–1.06	0.49
Volume	1.02	0.99–1.05	0.13
Recurrence	1.00	0.29–3.50	1.00
Pathology	0.40	0.08–1.91	0.25
Visual impairment duration	1.00	0.94–1.05	0.89
Tight adhesion	0.90	0.29–2.77	0.85

## Discussion

With the remarkable development of neurosurgery, the concept of protecting neurological function while treating diseases has become deeply rooted. IONM, as an important method to protect neurological function during surgery, has been widely used in various neurosurgical procedures. At present, EEEA is the optimal surgical approach for craniopharyngioma surgery without significant lateral invasion (11, 12). As the tumor is adjacent to the optic nerve and chiasm, the surgical removal of the tumor may lead to strain damage to the optic nerve and chiasm, and thus result in new or intensified deterioration of visual function post-operatively, which can significantly decrease the quality of life of the patients. VEP is a long latency-evoked potential recorded in the occipital region of the visual primary cortex after performing flash stimulation on the human eyes, and it can reflect the functional integrity of visual pathway. Accordingly, it is of great clinical importance to apply VEP monitoring to EEEA to protect the visual function of patients.

The main objective of this study was to determine the relationship between intraoperative P100 latency changes and post-operative visual deterioration. Although the relationship between the prolonged P100 latency and visual loss has been reported previously (13, 14); however, to our knowledge, this is the first large sample study on this issue. From the present findings, the incidence of post-operative visual deterioration was significantly higher in patients with prolonged post-operative P100 latency than in patients without prolonged P100 latency, the degree of prolonged post-operative visual deterioration of P100 latency was significantly higher in patients with deteriorated visual acuity than in patients without deterioration, and using a P100 latency prolongation degree of 8.61% as the critical threshold can refine the current warning criteria of VEP monitoring. This finding is of clinical significance, and can further reduce the incidence of post-operative visual deterioration. It is clear that a threshold value of 8.61% will be difficult to grasp in clinical practice, and the application of automatic recognition methods is needed in the coming era of precision medicine.

As early as 1973, Wright et al. first attempted to apply VEPs for intraoperative monitoring under general anesthesia, and the waveforms elicited were unstable due to the conditions at that time (15). In recent years, the development of total intravenous anesthesia techniques and light-emitting diode technology has led to the stable generation of VEP. To date, many clinical studies have explored and confirmed a good correlation between intraoperative VEP and post-operative visual function, but most of them are limited to the relationship between VEP amplitude changes and visual function (16–20), and VEP is reliable in predicting new visual damage at 50% alarm criteria (16, 20). Previous studies have paid less attention to P100 latency in VEP, and none of them could summarize the threshold for the degree of P100 latency in VEP prolongation (21). In the present work, data from 180 eyes in 90 patients were analyzed, and 8.61% was determined as the threshold for the degree of P100 prolongation for predicting post-operative visual deterioration. The incidence of post-operative visual deterioration was significantly higher in patients with a degree of P100 prolongation ≥8.61%. The results of this work may further refine the alarm criteria for VEP monitoring in predicting new visual impairment.

Visual-evoked potential is a routinely applied intraoperative optic nerve monitoring technique that can continuously monitor the integrity of the visual conduction pathway in real time and assess the status of intraoperative optic nerve function, which is done without interference to the operator. After intraoperative stimulation of the optic nerve by traction, cautery, and compression, the P100 latency will be prolonged. At this time, timely adjustment of the surgical strategy will enable some patients to have a certain degree of recovery of the P100 latency and no significant deterioration of post-operative visual acuity. Hayashi and Kawaguchi concluded that hypocapnia likewise leads to prolonged VEP latency, and therefore, hypocapnia is avoided as much as possible during total intravenous anesthesia procedures (22). Since P100 latency varies greatly among individuals, the degree of P100 latency prolongation was used as the judgment index, but there is still the possibility of false negatives and false positives. In this work, we found that the P100 latency was shortened in two eyes after surgery compared with the preoperative period, and the post-operative visual function still deteriorated, and a false negative result occurred. The reason for post-operative visual deterioration was analyzed as ischemia caused by vasospasm. After monitoring 100 cases by Sasaki et al. (16) and 53 surgeries by Kodama et al. (17) it was found that the sensitivity of using VEP amplitude to predict visual dysfunction was up to 87.5% and the specificity was 98.8%. These two studies found high specificity, but slightly lower sensitivity for monitoring VEP amplitude changes. By comparison, here we obtained a higher sensitivity (96.8%) and a slightly lower specificity (81.8%) in the VEP latency.

Our study has limitations. First, the sample size was still relatively small, and it is still unclear whether P100 delay can be used as an independent predictor. Secondly, our study was limited by retrospective nature. Future sample size expansion and prospective studies are needed to validate our results.

## Conclusion

This study revealed that intraoperative VEP monitoring is an effective method for preventing visual deterioration during EEEA. P100 latency in VEP prolongation of 8.61% can be used as a critical cutoff to predict post-operative visual deterioration.

## Data Availability Statement

The raw data supporting the conclusions of this article will be made available by the authors, without undue reservation.

## Ethics Statement

The studies involving human participants were reviewed and approved by the Ethics Committee of Beijing Tiantan Hospital. Written informed consent to participate in this study was provided by the participants' legal guardian/next of kin.

## Author Contributions

XT and HQ: study concept, design, and writing—review and editing. XT, XY, DG, and HY: data acquisition and analysis. XF, YJ, and JL: formal analysis and investigation. XT: writing—original draft preparation. HQ: funding acquisition. CL and HQ: supervision. All authors contributed to the article and approved the submitted version.

## Funding

This study was supported by the Beijing Municipal Science & Technology Commission (Grant No. Z191100006619087).

## Conflict of Interest

The authors declare that the research was conducted in the absence of any commercial or financial relationships that could be construed as a potential conflict of interest.

## Publisher's Note

All claims expressed in this article are solely those of the authors and do not necessarily represent those of their affiliated organizations, or those of the publisher, the editors and the reviewers. Any product that may be evaluated in this article, or claim that may be made by its manufacturer, is not guaranteed or endorsed by the publisher.
